# Versatile roles of innate lymphoid cells at the mucosal barrier: from homeostasis to pathological inflammation

**DOI:** 10.1038/s12276-023-01022-z

**Published:** 2023-09-11

**Authors:** Seungwon Ryu, MinYeong Lim, Jinwoo Kim, Hye Young Kim

**Affiliations:** 1 Department of Microbiology, Gachon University College of Medicine, Incheon, 21999 South Korea; 2 Laboratory of Mucosal Immunology, Department of Biomedical Sciences, Seoul National University College of Medicine, Seoul, 03080 South Korea; 3 Institute of Allergy and Clinical Immunology, Seoul National University Medical Research Center, Seoul, South Korea; 4 CIRNO, Sungkyunkwan University, Suwon, South Korea

**Keywords:** Innate lymphoid cells, Mucosal immunology

## Abstract

Innate lymphoid cells (ILCs) are innate lymphocytes that do not express antigen-specific receptors and largely reside and self-renew in mucosal tissues. ILCs can be categorized into three groups (ILC1–3) based on the transcription factors that direct their functions and the cytokines they produce. Their signature transcription factors and cytokines closely mirror those of their Th1, Th2, and Th17 cell counterparts. Accumulating studies show that ILCs are involved in not only the pathogenesis of mucosal tissue diseases, especially respiratory diseases, and colitis, but also the resolution of such diseases. Here, we discuss recent advances regarding our understanding of the biology of ILCs in mucosal tissue health and disease. In addition, we describe the current research on the immune checkpoints by which other cells regulate ILC activities: for example, checkpoint molecules are potential new targets for therapies that aim to control ILCs in mucosal diseases. In addition, we review approved and clinically- trialed drugs and drugs in clinical trials that can target ILCs and therefore have therapeutic potential in ILC-mediated diseases. Finally, since ILCs also play important roles in mucosal tissue homeostasis, we explore the hitherto sparse research on cell therapy with regulatory ILCs. This review highlights various therapeutic approaches that could be used to treat ILC-mediated mucosal diseases and areas of research that could benefit from further investigation.

## Immunology: the role of innate lymphoid cells

Cells of the immune system called innate lymphoid cells (ILCs) patrol the soft ‘mucosal’ tissues lining the respiratory, digestive and reproductive organs, where they can be involved in both the recovery from and development of disease. Researchers in South Korea led by Hye Young Kim at Seoul National University, review recent advances in understanding the biology of ILCs in mucosal tissue health and disease. ILCs are part of the innate immune system, which elicits immediate and general responses to infection and disease, rather than responses to specific foreign substances. The authors discuss physiological mechanisms that regulate ILCs, which could be targeted for future drug development. They also review the potential of existing drugs that could target ILCs to treat disease, especially inflammatory conditions, and the possible use of ILCs as cell-based therapies for various diseases.

## Introduction

Our bodies are constantly being challenged by pathogens and noxious stimuli, and mucosal tissues serve as the primary defense against these threats. This defense is mediated not only by the barrier function of the mucosal tissues but also by a sophisticated local immune system that connects with the systemic immune system. A key component of the local system is innate lymphoid cells (ILCs): Multiple studies have revealed that ILCs play important roles in tissue homeostasis and inflammation^1^. They are largely tissue-resident lymphocytes whose precursors settle in the tissues during organ development; once mature, they perform multiple effector functions that are often highly tissue-specific^1,2^. Although we now know that ILCs are present in almost all organs, the fact that ILCs were first discovered in the mucosa and are abundant in these tissues compared to other organs suggests that they play a particularly essential role in mucosal tissues.

ILCs lack specific antigen receptors such as T-cell receptors (TCRs) or B-cell receptors; rather, they recognize signals emitted by the tissue, particularly cytokines. They also have receptors for microbial products, nutrient components, lipid mediators, and neuronal transmitters^1^. Interestingly, ILC subsets closely resemble T-cell subsets in terms of the transcription factors that drive their activities and the cytokines they produce. Specifically, natural killer (NK) cells resemble cytotoxic T cells; type-1 helper ILCs (ILC1s) are similar to T-helper (Th)1 cells; group-2 ILCs (ILC2s) correspond to Th2 cells; and group-3 ILCs (ILC3s) are similar to Th17/22 cells. ILC1s and NK cells together form group-1 ILCs. They constitutively express the T-box transcription factor T-bet (encoded by *Tbx21*), which is essential for NK cell maturation and the early development of ILC1s^3^. However, the formation of NK cells and their cytotoxic functions also require the expression of Eomesodermin (Eomes)^4^. NK cells and helper ILC1s bear receptors for IL-12 and IL-18 and produce IFN-γ and TNF-α^5^. ILC2s express two transcription factors, namely, GATA-binding protein-3 (GATA3) and retinoic acid receptor-related orphan receptor (ROR) α (RORα). They bear the IL-33 receptor (IL-33R)^6^ and produce large amounts of type 2 cytokines (e.g., IL-5 and IL-13) as well as other cytokines such as amphiregulin, GM-CSF, and IL-9 ^7,8,9^. ILC3s are regulated by the RORγt and aryl hydrocarbon receptor (AHR) transcription factors, express receptors for IL-1 and IL-23, and secrete IL-17 and IL-22^10,11^. ILC3s are subdivided into three populations depending on their expression of CCR6 and natural cytotoxicity receptor (NCR, i.e., NKp46 in mice and NKp44 in humans). CCR6^+^ cells are designated lymphoid tissue inducer cells (LTis) and produce lymphotoxins (LTs). CCR6^-^ cells are further divided into NCR^+^ ILC3s and NCR^-^ ILC3s^12^.

ILCs are highly sensitive to tissue-intrinsic and -extrinsic signals, which result in transcriptional and epigenetic modifications that permit these cells to exert their wide array of effector functions; these functions include lymphoid organogenesis, regulating innate immune responses against insults to the mucosal tissue, and maintaining metabolic and immunological tissue homeostasis^13^. Here, we provide an overview of recent studies on the biology of ILCs in mucosal tissues, particularly the lung and intestine. Of particular interest are the tissue-specific characteristics and behaviors of ILCs. We also discuss what is currently known about potential ILC immune checkpoints that could be targeted therapeutically. Finally, we detail the drugs currently used or that could be repurposed to treat ILC-mediated diseases of mucosal tissues and the potential of cell-based therapies for these diseases.

## Tissue-specific distribution of ILCs

Most ILCs are tissue-resident lymphocytes: parabiosis experiments have shown that in adulthood, the vast majority of helper ILCs in various organs are derived from self-renewing ILC precursors (ILCps) that develop in the fetal liver during ontogeny or infancy and then migrate to emerging tissue^14^. These cells are defined as lineage- PLZF^+^ α4β7^+^ IL-7Rα (CD127)^+^ PD-1^+^ cells^15,16^. The remaining minority of tissue ILCs in adults are derived from ILCps that originate as hematopoietic stem cells in the bone marrow and then migrate to the tissue during adulthood^17^; this phenomenon is reflected by the fact that at any given time, 30–40% of the IL-18Rα^+^ ST2^−^ ILCps in the lung are located in the pulmonary vasculature and express migration-associated genes^18^. Despite their distinct origins, however, the tissue-resident ILCps (identified as lineage- RORα^+^ Thy1^+^ CD127^+^ IL-18Rα^+^ ST2^−^ cells) derived from the fetal liver closely resemble those derived from bone marrow in terms of phenotype and differentiation potential^16^.

The homing of both fetal liver- and bone marrow-derived ILCps from the circulation into mucosal and non-mucosal tissues is partially mediated by integrin α4β7, which is expressed by ILCps^19,20^, and its binding partners on endothelial cells, namely, MAdCAM-1 (mucosal addressin cell adhesion molecule 1) and VCAM-1 (vascular cell adhesion protein 1)^21,22^. However, many other factors also shape ILCp homing to tissues, including chemoattractant receptors^23–25^. For example, CXCR6 on ILCps partially determines their retention in both the bone marrow in adults and the fetal liver during embryonic life^23^. Moreover, some homing-related factors promote tissue-tropic ILCp homing; for example, IL-33, which is secreted by lung epithelial cells when they are damaged (e.g., by *Nippostrongylus brasiliensis* infection of the lungs), induces the egress of ILC2ps from the adult bone marrow and their trafficking to the lung. The mechanism involves IL-33 downregulating CXCR4 in bone marrow ILCps, which normally enforces retention of these cells in the bone marrow^18,26^. In addition, the differentiation of tissue ILCps into ILC1s, ILC2s, or ILC3s appears to be largely driven by local signals; for example, although IL-18Rα^+^ST2^−^ lung-resident ILCps normally differentiate into ILC2s, *Mycobacterium tuberculosis* infection can induce their differentiation into IFN-γ-producing ILC1-like cells^27^.

As a result of these local factors, ILC1s are largely concentrated in the intraepithelial layer of the intestine^28^, while ILC2s predominate in the lung, skin, and white adipose tissue, and ILC3s mainly reside in the lamina propria (LP), cryptic patches, and Peyer’s patches of the intestinal tract^29,30^. This tissue-specific localization of ILCs in peripheral tissues may play important roles in the homeostasis and inflammation of these tissues and the body.

## Pathogenic roles of ILCs in mucosal tissues

While non-mucosal tissues also bear ILCs, they are particularly abundant in mucosal tissues and often demonstrate significant changes in mucosal diseases^13^. Therefore, this review will focus on the most studied lung and intestinal ILCs.

### ILCs in the lung

**Fig. 1:**
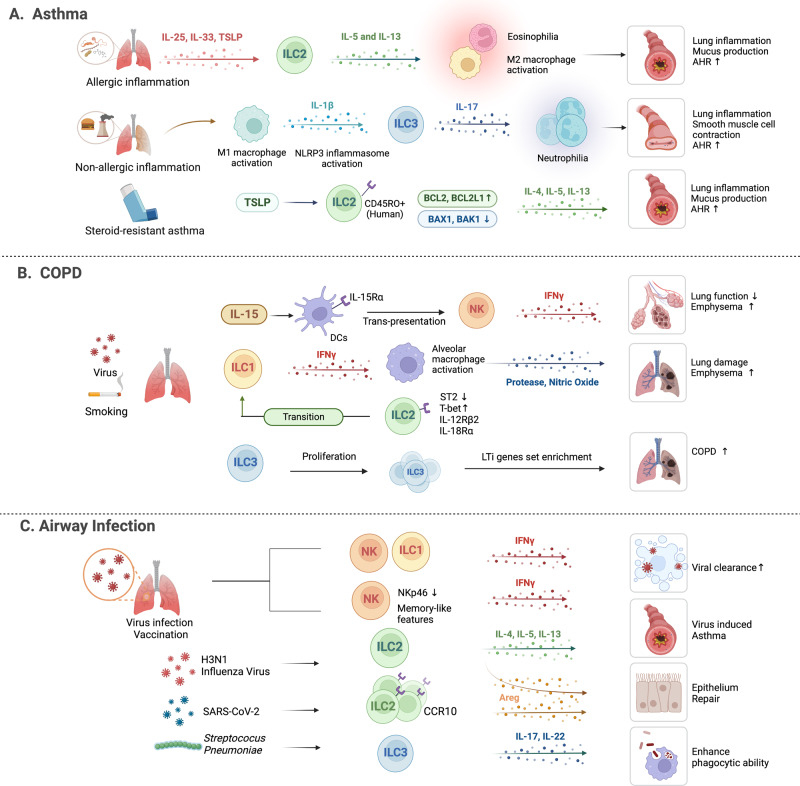
The function of ILCs in respiratory diseases. ILCs play a significant role in respiratory diseases, including asthma, COPD, and respiratory infections. **A** In allergic asthma, alarmins released by allergen-damaged lung epithelium induce activation of ILC2s. ILC2s recruit other immune cells, including eosinophils and alternatively activated M2 macrophages, leading to the induction of AHR and airway Inflammation. In non-allergic asthma caused by obesity and pollutants, IL-1β is induced by lung macrophages and activates lung ILC3s. **B** In COPD, the roles of ILC1s and ILC3s are better understood than that of ILC2s. IFN-γ-producing ILC1s induce alveolar M1 macrophages to release elastolytic proteases and nitric oxide, resulting in emphysema generation. NK cells have been shown to have enhanced cytotoxic function, which is driven by IL-15 secreted by dendritic cells. **C** Viral infection and long-term smoking convert lung ILC2s into ILC1-like populations. ILCs can mediate versatile effects in respiratory infection. NK cells and ILC1s contribute to viral clearance. However, ILC2s induce AHR and airway inflammation via type 2 cytokines and epithelium recovery through amphiregulin. ILC3s also play both protective and inflammatory roles in infection mediated by IL-17A and IL-22 secretion.

The lung comprises the lower respiratory tract, including the bronchi, bronchioles, and alveoli^31^. The epithelial lining of the lung forms an essential barrier that not only exchanges gas but also senses and responds to external insults^31^. In mice, ILC2s are the predominant ILC population in the respiratory tract, whereas ILC3s are the most abundant in humans^32^. Respiratory diseases such as asthma, chronic obstructive pulmonary disease (COPD), and infection can be associated with dramatic and dynamic changes in the composition and functions of ILC subsets in the lung (Fig. 1). Below, we discuss what is known about the roles of ILCs in lung diseases.

#### Asthma

Asthma is caused by repeated exposure to specific allergens or host/environmental factors that induce breathing difficulty, coughing, and wheezing. These symptoms are directly caused by airway hyperreactivity (AHR), mucus overproduction, and airway remodeling^33^. Asthma is a heterogeneous disease composed of many endotypes. Of these, allergic asthma is the most common. It is triggered by inhaled allergens that damage the lung epithelium, which then initiates a cascade of pathogenic events. Specifically, the damaged epithelium releases alarmins, including IL-33, IL-25, and TSLP, which activate ILC2s^34^^,^^35^: these cells then recruit other immune cells, including eosinophils and alternatively activated M2 macrophages, which induce AHR and airway inflammation^36^. The key roles of alarmins and ILC2s in allergic asthma have been shown by multiple studies. For example, a genome-wide association study showed that asthma is associated with polymorphisms in the genes that encode IL-33 and IL-33 receptor (ST2)^37,38^. Moreover, the peripheral blood and induced sputum of patients with asthma contain higher IL-33 levels and ILC2 frequencies than those of control subjects^39–41^.

Another common asthma endotype is non-allergic asthma, which affects up to one-third of asthma patients^42^. One form is induced by obesity, which may be mediated by high-fat diet-induced activation of lung macrophages that produce IL-1β and thereby activate lung ILC3s^43^. Another form of non-allergic asthma is generated by repeated exposure to environmental pollutants (particulate matter, diesel exhaust particles, ozone, and carbon nanotubes); it may also be associated with ILC-induced airway inflammation since repeated exposure to pollutants increases the numbers of pulmonary ILC2s and their IL-5 and IL-13 production^44,45^.

ILCs are suspected to be one of the causes of steroid-resistant asthma. For example, one study reported that the levels of circulating ILC2s from steroid-resistant asthma patients increase after steroid treatment; moreover, circulating ILC2s from healthy humans are related to steroid resistance mediated by high expression of the anti-apoptotic genes *BCL2* and *BCL2L1*. The ratio of the proapoptotic genes *BAX1* and *BAK1* to the anti-apoptotic gene *BCL2* was significantly decreased in ILC2s^46^. Another study reported that asthma patients bear more CD45RO^+^ ILC2s (which correspond to murine inflammatory ILC2s) in their sputum than controls and that the frequency positively correlates with steroid resistance^47^. In addition, sputum ILC2s from asthma patients demonstrate steroid resistance, and their numbers are positively correlated with the sputum levels of TSLP^48^. The outcome of β2-adrenergic receptor (β2AR) agonist treatment also shows a negative correlation with ILC2 frequency and cytokine production, in both murine models and asthma patients^49^. These results together suggest that ILCs contribute to the development of asthma.

#### COPD

COPD is an irreversible chronic lung disease characterized by long-term breathing problems and obstructed airflow^50^. Although the role of ILCs in COPD is less well-researched than that in asthma, a recent study reported that patients with COPD have higher frequencies of ILC1s in the blood than healthy controls and that patients with COPD have higher IFN-γ-producing ILC1 frequencies in the lung than control subjects^51^. The mechanism by which ILC1s induce COPD may involve their production of IFN-γ: it has been shown that this can induce alterations in the pulmonary protease and protease inhibitor balance which destroy the lung tissue and thereby generate emphysema^52,53^. In addition, NK cells, which also belong to the group-1 ILCs, acquire a phenotype in COPD that not only heightens the ability of these cells to kill autologous lung cells in vitro but also is correlated with the reduction in lung function and emphysematous destruction in COPD in vivo^54^. The enhanced killing functions of these cells appear to be driven by trans-presentation of IL-15Rα secreted by lung dendritic cells (DCs)^55^. Notably, two risk factors for COPD, namely, viral infection and long-term smoking, have been observed to convert lung ILC2s into ILC1-like populations that express IL-12Rβ2, IL-18Rα, T-bet, and IFN-γ both in vitro and in vivo^56,57^. Moreover, smoke exposure induces lung epithelial cells to secrete IL-33 but also alters lung immune reactions to this cytokine: resident ILC2s demonstrate decreased ST2 expression and type 2 activity, whereas macrophages and NK cells display augmented ST2 expression and greater production of type-1 proinflammatory cytokines^58^. There is some limited evidence suggesting that ILC3s also promote COPD pathogenesis: compared to those from control subjects, lung tissues from COPD patients have higher NCR- ILC3 frequencies^59^ and are enriched for the expression of genes related to the LTi subset of ILC3s^60^.

Thus, risk factors for COPD upregulate ILCs, these cells bear pathological characteristics, and the numbers of these cells correlate with disease severity. These findings suggest that ILCs mediate COPD.

#### Infection

Respiratory infections are classified as upper and lower respiratory infections^61^. Studies suggest that while ILCs generally protect the lung from respiratory viral infections, they can also mediate the pathogenic effects of these infections^62^. With regard to the protective roles of ILCs, NK cells and ILC1s are well known to contribute to immune responses against viruses by secreting IFN-γ. Interestingly, influenza virus infection or vaccination generates memory-like NK cells. Thus, subsequent challenges are associated with a rapid increase in IFN-γ but the downregulation of NKp46-expressing NK cells^63^. In contrast, ILC2s play both pathogenic and beneficial roles in respiratory viral infections^7,64^. On the one hand, influenza A virus (H3N1) can rapidly induce AHR by activating ILC2s: these cells produce IL-5 and IL-13, which in turn induce the accumulation of eosinophils in the lung^65^. On the other hand, ILC2s promote recovery from virus-induced AHR: a recent study reported that the recovery of patients with severe COVID-19 correlates strongly and positively with the frequency of CCR10^+^ ILC2s in their blood^66^. Moreover, hospitalized COVID-19 patients have fewer amphiregulin-producing ILC2s in their blood than COVID-19 outpatients and uninfected control subjects^67^. In addition, when ILC2s are depleted in mice infected with the influenza A virus, the mice demonstrate further reductions in lung function, airway epithelial integrity, and respiratory tissue remodeling; notably, these effects are reversed by intraperitoneal administration of amphiregulin^7^. Thus, ILC2s may promote lung homeostasis by producing amphiregulin. Finally, lung ILC3s play a protective role mediated by their IL-17 and IL-22 secretion in *Streptococcus pneumoniae*-infected mice^68^.

Thus, ILCs generally act to protect the lungs from infection, although they can also promote the damaging effects of respiratory infections in some cases.

### ILCs in the intestine

**Fig. 2:**
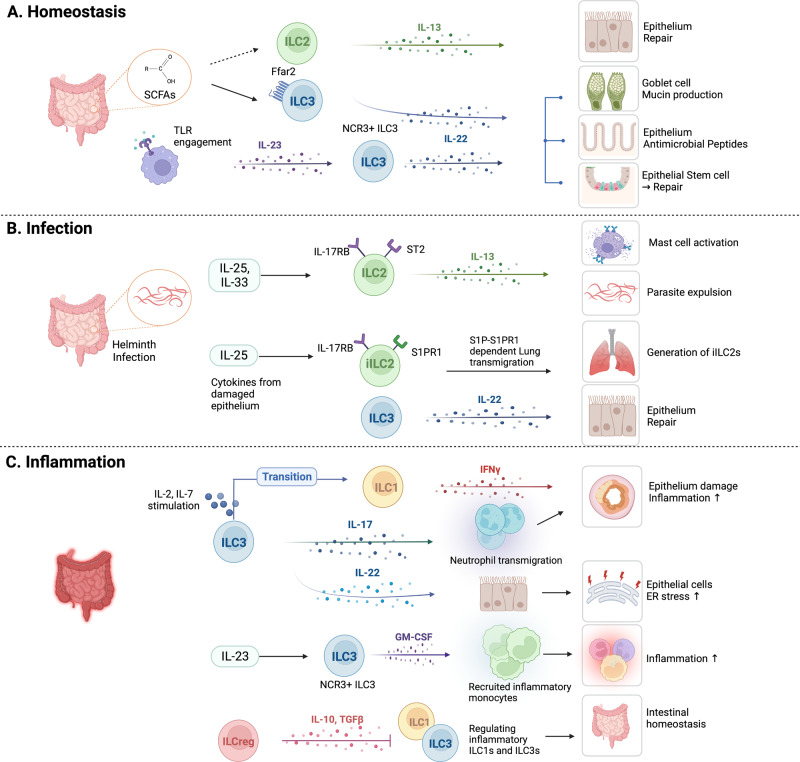
Role of ILCs in the intestine. ILCs also play significant roles in maintaining intestinal homeostasis and in disease conditions. **A** ILC3s contribute to intestinal homeostasis by secreting IL-22, preventing epithelial barrier disruption. Additionally, IL-22 induces mucin production in goblet cells and antimicrobial peptide production in epithelial cells. ILC2s contribute to intestinal homeostasis by stimulating epithelial regeneration through IL-13 production. **B** During intestinal parasite infection, intestinal ILC2s play important roles in anti-parasitic mechanisms. IL-25 and IL-33 released by parasite-damaged epithelium cause the expansion and activation of local ILC2s, ultimately resulting in parasite expulsion. IL-22 secreted by ILC3s in a parasitic environment promotes intestinal stem cell-mediated epithelial regeneration. **C** ILCs also play a pathogenic role in intestinal inflammation mediated by the production of IL-17A in ILC3s and IFN-γ in ILC1/3s. Regulatory ILCs (ILCregs) control inflammatory ILC1s and ILC3s through IL-10 and TGF-β1 production in the intestines. Although IL-22 produced by ILC3s acts as a protective factor in maintaining intestinal homeostasis, it can also promote acute colitis by endoplasmic reticulum (ER) stress in epithelial cells.

The gastrointestinal (GI) system consists of the stomach, small intestine (i.e., the duodenum, jejunum, and ilium), and large intestine or colon. Moving away from the lumen, the intestine layers consist of the mucosa, submucosa, muscularis propria, and serosa^69^. The mucosa, in turn, consists of the intestinal epithelium, the LP, and the muscularis mucosa. The mucosa of the small intestine also contains multiple Peyer’s patches and cryptopatches, a unique secondary lymphoid organ. The intestinal epithelium, LP, Peyer’s patches, and cryptopatches are the main sites that contain ILCs; these are also the sites where immune cells act in intestinal immune responses, which are highly sophisticated and enable the GI system to continue absorbing nutrients in an environment constantly exposed to pathogens and commensal microorganisms. Notably, the intestinal epithelium and LP of the murine small and large intestines demonstrate some heterogeneity in terms of ILC composition: specifically, the LP in the lower intestine is occupied by ILC2s, whereas ILC3s predominate in all other intestinal compartments^70^. As will be detailed below, intestinal ILC3s play crucial homeostatic roles in the intestine but can also contribute to inflammatory bowel diseases (IBDs); intestinal ILC2s are important for expelling worms from the gut; and the intestine also contains regulatory ILCs that may play essential roles in intestinal homeostasis (Fig. 2).

#### Role of ILC3s in intestinal homeostasis

ILC3s play essential roles in intestinal homeostasis^71,72^. In particular, they help prevent the disruption of the epithelial barrier, which can lead to infection and intestinal disorders such as IBDs. This role of ILC3s is mediated by their secretion of IL-22, a key cytokine in intestinal homeostasis, while other cell types can produce IL-22, RORγt^+^ ILC3s are the main producers of IL-22 in the intestine^73,74^. IL-22 induces goblet cells to produce mucin proteins that protect the epithelial barrier^75^. Moreover, IL-22-producing ILC3s (especially NCR^+^ ILC3s) induce STAT3 phosphorylation in intestinal stem cells, which permits the repair of damaged tissue^73,76–78^. In addition, when the intestine is inflamed, IL-22-producing ILC3s engage in patrolling behavior that directly prevents epithelial cell death^79^. IL-22 also induces intestinal epithelial cells to produce antimicrobial peptides, including RegIIIβ and RegIIIγ, thus preventing pathogenic bacteria from inducing intestinal lesions^74^.

The production of IL-22 by ILC3s in the intestine appears to be driven by direct and indirect interactions between ILC3s and commensal microbiota^76^. The direct interactions may be mediated by intrinsic receptors on intestinal ILCs, such as free fatty acid receptor 2 (Ffar2). Ffar2 is a G-protein-coupled receptor that recognizes bacterial metabolites, specifically short-chain fatty acids^80^. The indirect interactions may involve signals from other cells, such as myeloid cells. When the Toll-like receptors on these cells are engaged by bacterial proteins, they produce IL-23, which activates ILC3s to produce IL-22^81,82^

#### Role of ILC3s and ILC1s in intestinal diseases

ILC3s also play a pathogenic role in the intestines and promote IBDs. This role is largely mediated by their production of IL-17 and IFN-γ^83–85^, while IFN-γ production is a hallmark cytokine of ILC1s, ILC3s bear some plasticity that allows them to express IFN-γ when stimulated by combinations of cytokines such as IL-2 + IL-7^86^. In addition, IL-22-expressing ILC3s can be converted into IFN-γ-expressing ILC1s in certain circumstances^87^. IL-17 induces massive neutrophil transmigration, which disrupts epithelial junctions and increases epithelial permeability, while excessive production of IFN-γ causes epithelial cell damage that exacerbates the inflammatory reaction^87^. ILC3s may also contribute to IBDs by producing large amounts of GM-CSF, which recruits and maintains inflammatory monocytes; this role is mediated by NCR^+^ ILC3s^88^. The inflammatory monocytes induced by NCR^+^ ILC3s promote tissue damage and dysbiosis and generate a positive feedback loop that amplifies inflammation^88^. NCR^-^ ILC3s can also promote IBDs: the intestinal inflammatory environment can cause NCR^+^ ILC3s to convert into NCR^-^ ILC3s that also secrete GM-CSF. This cytokine then recruits neutrophils, which in turn damage the tissue by secreting large amounts of proinflammatory cytokines and matrix metalloproteinases^89^.

Notably, although IL-22-producing ILC3s play a key protective role in GI homeostasis, they can also promote acute colitis. This may be mediated by IL-22-dependent endoplasmic reticulum stress in colon epithelial cells^90^. The pleiotropic behavior of intestinal ILC3s is partly explained by their different modes of action following disease progression. Thus, in the context of acute colitis, ILC3s are activated by TNF-like ligand 1 A (TL1A) from CX3CR1^+^ mononuclear phagocytes to produce IL-22. However, TL1A induces expression of OX40L, a costimulatory molecule, on ILC3s to activate IFN-γ^+^ CD4^+^ T cells, which exacerbates colitis during chronic T-cell-mediated colitis^91^.

#### Role of ILC2s in the Intestine

Intestinal ILC2s play key roles in eliminating intestinal parasites, including *N. brasiliensis*^92^. This is mediated by their production of type 2 cytokines, particularly IL-13, which stimulates mast cells to produce proteases and induces parasite expulsion^93,94^. Although IL-13 is secreted by various cell types, including Th2 cells, ILC2s are the main producers of IL-13 in parasite infections, especially during the early phase. IL-13 production by ILC2s is induced by the release of IL-25 and IL-33 by parasite-damaged epithelial cells: IL-25 activates and expands local IL-25R (IL-17RB)-expressing ILC2s, termed inflammatory ILC2s (iILC2s), which are abundant in the intestine, while IL-33 activates and expands the small population of IL-33R (ST2)-expressing ILC2s in the intestine, termed natural ILC2s (nILC2s). Both iILC2s and nILC2s participate in the expulsion of *N. brasiliensis*^93,94^. The predominance of iILC2s and the small nILC2 population in the intestine under homeostatic conditions is different from the case in other organs, which are dominated by nILC2s^95^. Notably, IL-25-responsive intestinal iILC2s can migrate to the lungs after *N. brasiliensis* infection: this migration event depends on sphingosine-1-phosphate (S1P) signaling and contributes to the systemic anti-helminth response^96^.

#### Role of regulatory ILCs in the intestine

Along with group-1–3 ILCs, the ILC family also contains a fourth ILC type, namely, regulatory ILCs (ILCregs). They correspond to regulatory T cells (Tregs) in the adaptive immune system. These ILCregs have been found in the intestine^97^. They produce IL-10 and TGF-β1, which are responsible for controlling various intestinal inflammatory conditions, possibly by regulating inflammatory ILC1s and ILC3s^97^. Studies on this novel ILC subset are lacking, but regulatory ILCs deserve continued attention since they may serve as critical mediators of the tissue immune response.

## Recent advances regarding the regulation of ILCs by immune checkpoint molecules

**Fig. 3:**
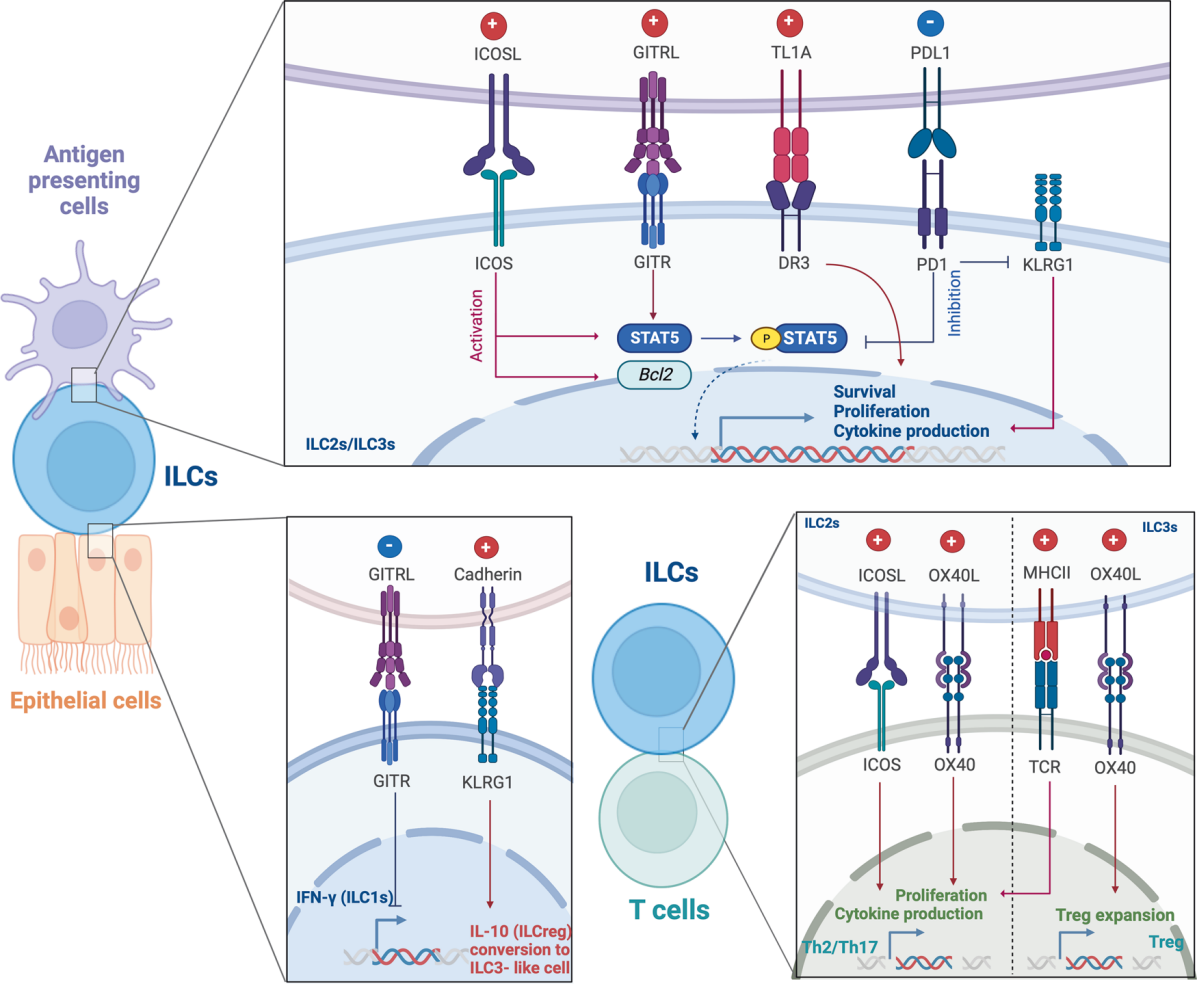
Immune checkpoint molecules in ILCs. Many different immune checkpoint molecules control both ILCs and interactive cell responses. ICOS-mediated signals regulate ILC function by controlling the expression of *Bcl2* and the phosphorylation of STAT5. Both ICOS and ICOSL are expressed in ILC2s, resulting in the production of type 2 cytokines and the expansion of regulatory T cells. GITR enhances ILC2 function by increasing the production of IL-5, IL-13, and IL-9 via the STAT5 pathway. ILCs also express OX40L, which triggers the effector function of OX40^+^ regulatory T cells. All ILC subsets bear DR3 on their surface, which promotes cytokine production and proliferation. PD-1 acts as a restrictive molecule in ILCs, downregulating inflammatory effects and KLRG1 on ILC2s. The role of KLRG1 in all ILC subtypes remains unclear, but studies have shown that KLRG1 appears to mediate the protective function of ILC2s.

The job of immune cells is to mount appropriate immune responses to immunological threats, but persistent overreactions can lead to tissue damage and autoimmunity. Above, we showed that ILCs can shift from their homeostatic protective roles to become pathogenic. However, due to the relatively recent discovery of ILCs, the mechanisms that could block or mediate these shifts are poorly understood. In contrast, much more is understood about the mechanisms related to the adaptive counterparts of ILCs, namely, T cells. Considerable research on these mechanisms has led to the identification of key immune checkpoint molecules that can be targeted therapeutically to successfully contain T-cell-mediated diseases. These observations have led to the following question: what are the mechanisms that control ILC reactivity? Given that ILCs are innate counterparts of T cells, can the immune checkpoints in T-cell immune responses also play similar roles in ILCs? Since immune checkpoints may play important roles in ILC-mediated tissue homeostasis, inflammatory responses, and antitumor responses, these questions have aroused considerable interest in the last few years. Here, we will review the current understanding regarding the contribution of immune checkpoint molecules to ILC function in mucosal tissues (Fig. 3).

### ICOS-ICOSL

Inducible T-cell costimulator (ICOS) is a member of the CD28 receptor family^98^ and is highly expressed by activated T cells and regulatory T cells^99,100^. The ICOS ligand (ICOSL) is mainly expressed by DCs, and the binding of ICOSL to ICOS on T cells enhances their differentiation and effector functions^101,102^. Studies show that ILC2s express high levels of ICOS in the steady state, and ICOS-mediated signals regulate ILC2 functions^103–105^. Specifically, ICOS-deficient mice have reduced ILC2 frequencies and IL-13 production in the lungs and small intestine due to decreased expression of the anti-apoptotic molecule *Bcl2*^103,104^. As a result, ICOS-deficient mice do not demonstrate ILC2-dependent lung inflammation in response to intranasal IL-33^104^ or papain^105^ administration. Notably, Kamachi et al. showed that while lung ILC2s may interact with ICOSL-expressing DCs in lungs with allergic inflammation^105^, Mazzi et al. reported that ILC2s express both ICOS and ICOSL and that ILC2-intrinsic ICOS:ICOSL interactions promote ILC2 cytokine production and survival *via* STAT5 signaling^104^. Most studies on ICOS in ILCs have focused on ILC2s, but ICOS may affect the functions of ILC3s. Iwanaga et al. showed with single-cell RNA-sequencing analysis that the lungs of *Klebsiella pneumoniae*-infected *Rag2*^*–/–*^ mice contain distinct clusters of *Il17a*^+^*Il22*^+^*Icos*^+^ ILC3s that are critical for host defense^106^.

Thus, ICOS signaling serves as a key regulator of lung ILC2 survival and asthma-related cytokine production. It may also mediate protective lung ILC3 functions. However, the mechanisms involved and whether these signals participate in diseases other than allergic asthma and infection remain unknown.

### GITR-GITRL

The glucocorticoid-induced TNFR-related (GITR) protein is encoded by *Tnfrsf18* and is a member of the TNFR superfamily^107^. It is stimulated by the GITR ligand (GITRL), mainly expressed by antigen-presenting cells and endothelial cells^108^. Recent studies have reported that ILCs express GITR^109,110^. Murine ILC1s express moderate levels of GITR, and influenza virus infection upregulates GITR expression on ILC1s, thereby impairing the host antiviral response by reducing their IFN-γ secretion^111^.

Unlike ILC1s, ILC2s express high levels of GITR^109,112^. Nonetheless, GITR expression levels on ILC2s are associated with asthma: RNA-sequencing analysis of lung ILC2s from *Alternaria*-treated mice showed that the expression of GITR in ILC2 populations correlated strongly and positively with their expression of ILC2 signature molecules (*Il1rl1* (encoding ST2)) and type 2 cytokines (*Il5* and *Il13*)^112^. Moreover, when *Gitr*^−/−^ mice were administered papain or IL-33, they demonstrated fewer lung ILC2s, less eosinophilia, and thus lower inflammation^110^. Similar results were obtained when GITRL was blocked by GITR-hFc injection before mice were administered papain intranasally. GITR appears to promote ILC2 functions by acting synergistically with ST2: ligation of both receptors causes ILC2s to produce IL-9 *via* STAT5 phosphorylation. The resulting IL-9 then acts on ILC2s in an autocrine manner to further augment their IL-5 and IL-13 production^110^. These results show that GITR could serve as an immune checkpoint for activated ILC2s. Thus, targeting GITR may be an attractive new therapeutic strategy for ILC2-mediated allergic and inflammatory diseases.

### OX40-OX40L

OX40 is a member of the TNFR superfamily and is expressed by activated CD4^+^ and CD8^+^ T cells^113^, while the OX40 ligand (OX40L, CD252, encoded by *Tnfsf4*) is expressed by antigen-presenting cells such as DCs and B cells. The engagement of OX40 sustains T-cell proliferation and survival^113^. Interestingly, ILCs express OX40L rather than OX40. Moreover, multiple studies have shown that OX40L-expressing ILCs can regulate the responses of T cells as antigen-presenting cells do^114–116^.

These OX40L-related T-cell regulatory roles include the ability of lung ILC2s to promote local T-cell responses to infections and allergens^114–116^, as shown by the fact that lung ILC2s express high levels of OX40L after intranasal administration of papain or IL-33 and promote the expansion of OX40-expressing Th2 cells. Moreover, specifically deleting OX40L in ILC2s (*Il7raCre*^*+/+*^*Tnfsf4*^*fl/fl*^ mice) blocked the effective Th2 and Treg responses induced by *N. brasiliensis* infection or allergen exposure^114^. In addition, when mice are infected with respiratory syncytial virus, their lung CD4^+^ T cells and ILC2s express high levels of OX40 and OX40L, respectively. OX40/OX40L interactions were further shown to promote CD4^+^ T-cell cytokine production in an ILC-dependent manner^115^. Similarly, when OX40L^+^ lung ILC2s and CD4^+^ T cells from naïve mice were adoptively transferred into naive *Il7ra*^*−/−*^ mice (which lack ILC2s and CD4^+^ T cells) that were then exposed intranasally to OVA and an adjuvant protease (bromelain), robust OVA-specific type 2 cytokine production and airway inflammation ensued. However, the transfer of either cell population alone did not have these effects^116^. These findings suggest that OX40L expression in ILC2s is critical for inducing OX40-expressing T-helper cells to produce type 2 cytokines.

The OX40L-related T-cell-regulating roles of ILCs are also observed in the intestine. All ILC3 subsets in the intestine express high levels of OX40L, particularly NCR^−^ ILC3s. OX40L expression by ILC3s appears to contribute to intestinal homeostasis^91,117^. Specifically, ILC3s and Tregs colocalize in the cryptopatches of the intestine, and *Tnfsf4*^*−/−*^*Rag1*^*−/−*^ mice (which lack T and B cells and OX40L expression) demonstrate expansion of intestinal Tregs when they are reconstituted with wild-type Tregs and ILC3s. However, when OX40L-deficient ILC3s are transferred along with wild-type Tregs, the Tregs do not demonstrate expansion in the intestine^117^. Since Tregs play key roles in suppressing inflammation, the crosstalk between Tregs and ILC3s in mucosal tissues *via* OX40-OX40L signaling is likely to be critical for intestinal homeostasis^117^. The importance of OX40L in intestinal homeostasis was also demonstrated by the study of Castellanos et al., which was mentioned above^91^. This study reported that TL1A expressed in CX3CR1^+^ mononuclear phagocytes could contribute to intestinal homeostasis by acting on OX40L of ILC3s and upregulating IL-22 production. Notably, this study also showed that the phagocyte-TL1A-OX40L axis mediates the ability of MHCII^+^ ILC3s to activate the antigen-specific T cells that drive chronic T-cell colitis^91^. Thus, OX40L expression in ILC3s can upregulate both Tregs and pathogenic T cells, thus driving not only intestinal homeostasis but also intestinal inflammation.

The role of OX40L in ILC1s is currently unknown. Nonetheless, the data for ILC2s and ILC3s suggest that OX40L is an important ILC-mediated immune checkpoint molecule, albeit one that can promote both homeostasis and disease depending on the circumstances.

### DR3-TL1A

Death receptor 3 (DR3) is encoded by *Tnfrsf25* and is a member of the TNFR superfamily. It is expressed by various immune cells, including T cells and macrophages^118,119^. The TNF family cytokine TL1A (TNF-like cytokine 1 A) appears to be the only DR3 ligand in inflamed tissues, including the lung and gut^120^. When T cells bind to TL1A *via* DR3, their cytokine production is stimulated^121^.

Recent studies have shown that all ILC subsets also bear DR3 on their surface and that this promotes cytokine production and proliferation^122–124^. In particular, TL1A binding to DR3 on human and murine ILC2s promotes expansion, survival, and functions^124^. Moreover, genetic overexpression of TL1A or exogenous TL1A administration activates lung ILC2s in vivo. In addition, DR3^−/−^ mice cannot elicit the lung ILC2 responses induced by intranasal papain challenge^124^. A study on patients with allergic asthma also supported the notion that TL1A interactions with DR3 on ILC2s may contribute to this disease: allergen challenges not only increased the TL1A levels in the airways but also elevated DR3 expression in lung ILC2s^125^. Thus, the TL1A-DR3 axis could contribute to allergen-induced airway inflammation by inducing ILC2s to secrete type 2 cytokines.

Intestinal ILC3s also express DR3 at high levels, and this expression may mediate their pathogenic activities in IBDs^126^. Colitis is exacerbated by injection of an agonistic anti-DR3 antibody; the colitis-worsening effects of the antibody are not observed in mice that lack ILC3s, which suggests that DR3-expressing ILC3s play a key role in initiating colitis. This effect may be mediated by ILC3 production of GM-CSF, which is upregulated by the agonistic antibody. Notably, this study showed that neutralization of TL1A by soluble DR3 ameliorates colitis, which suggests that DR3-expressing ILC3s may directly recognize TL1A^126^.

Thus, the limited studies to date suggest that DR3-mediated activation of ILC2s and ILC3s can lead to pulmonary and intestinal inflammation, respectively. DR3 is therefore a candidate ILC checkpoint molecule.

### PD-1-PD-L1

Programmed cell death-1 (PD-1) is a member of the immunoglobulin gene superfamily and the most representative inhibitory T-cell checkpoint molecule. It is a receptor that was originally identified in dying activated T cells^127^, and many tumors have been found to overexpress its ligand (PD-L1), thereby evading cytotoxic T cells^128^. Moreover, HIV-specific CD8^+^ and CD4^+^ T cells overexpress PD-1, which blocks their antiviral functions^129^. These findings have led to intensive research on the mechanisms by which the binding of PD-1 to PD-L1 regulates T cells in cancer and chronic infection^129,130^.

PD-1 was recently shown to participate in ILC development and functions as well. Specifically, it plays an important role during the development of all three ILC subsets since the adoptive transfer of PD-1^+^ ILCps into *Rag2*^*−/−*^*Il2rg*^*−/−*^ mice (which lack T and B cells, and ILCs) induces reconstitution of ILC1, ILC2, ILC3 and a small number of conventional NK cells but not T and B cells in the mice^131^. Moreover, although PD-1 deficiency does not significantly affect ILCp frequencies in the bone marrow or the development of mature ILCs in homeostatic conditions^132,133^, PD-1 is upregulated on lung ILC2s in lung inflammation, and ILC3s express functional PD-1 in the murine intestine^130^ human decidua^134^, and pleural effusions of patients with tumors^135^ . The role of PD-1-expressing ILCs in antitumor immunity is currently being investigated extensively. Since this topic has been addressed thoroughly by other reviews^136,137^, we will focus here on the roles that PD-1-expressing ILCs play in mucosal diseases other than cancer.

Lung ILC2s express PD-1 at high levels, and this expression is further increased by influenza infection and asthma-inducing intranasal stimulation with papain^131^, IL-33, house dust mite extract, or *Alternaria* extract^138^. In one study, a PD-1 agonist reduced lung ILC2s and ameliorated ILC2-mediated asthma; in this experiment, *Rag2*^*−/−*^*Il2rg*^*−/−*^ mice were reconstituted with ILC2s from human peripheral blood, treated with the agonist, and then challenged intranasally with IL-33 or house dust mite or *Alternaria* extract^138^. Moreover, PD-1-deficient *Pdcd1*^*−/−*^ mice display exacerbated IL-33-induced AHR and lung inflammation^138^. Thus, similar to its role in T cells^139^, PD-1 expression appears to regulate inflammatory lung ILC2s^132,138^.

The mechanism by which PD-1 restricts ILC2 functions may involve its ability to downregulate killer cell lectin-like receptor G1 (KLRG1) on ILC2s, which (as detailed below) promotes ILC2 effector responses^132^. This role of PD-1 is reflected by the fact that PD-1-deficient mice expel *N. brasiliensis* better than wild-type mice, and PD-1 deficiency is associated with high KLRG1 expression by lung ILC2s. Moreover, the adoptive transfer of KLRG1^+^ lung ILC2s from PD-1-deficient mice into mice lacking T cells and ILCs greatly increases worm expulsion^132^. PD-1 may also restrict ILC2s by shaping their metabolism: PD-1-deficient ILC2s display a metabolic shift toward glycolysis, glutaminolysis, and methionine catabolism, which is associated with increased effector function and survival^138^. Moreover, PD-1 may act by limiting STAT5 phosphorylation in ILC2s: blocking PD-1 or its downstream signaling molecule SHP1/2 significantly increases STAT5 phosphorylation in ILC2s^132^. Thus, PD-1 appears to be an inhibitory ILC2 checkpoint, and PD-1 agonists may be new ILC-specific therapies for asthma and allergy.

As mentioned above, ILC3s in the mouse intestine^131^ and human decidua^134^ express functional PD-1. The role of PD-1 in intestinal ILC3s has not been studied. However, Vacca et al. showed that in early pregnancy, ILC3s in the human decidua express PD-1, while invading trophoblasts express PD-L1. The PD-1/PD-L1 interactions regulate the production of cytokines by ILC3s, including IL-22, IL-8, and TNF-α. This suggests that PD-1 regulates the activities of ILC3s at the feto-maternal interface^134^.

The role of PD-1 expression in ILC1s is unknown. Further studies on the potential of PD-1 to act as an ILC checkpoint protein in ILCs other than ILC2s are warranted.

### KLRG1

KLRG1 is a C-type lectin expressed by many immune cell types, including NK cells and ILC2s^140,141^. However, blood-, tonsil-, and lung-resident ILC2s express significantly higher constitutive levels of KLRG1 than other ILC subsets^142^. Notably, although KLRG1 deficiency does not affect overall lung ILC2 frequencies or ST2 expression under homeostatic conditions or papain-induced asthma^143^, KLRG1 appears to be a marker for lung iILC2s. These inflammatory ILC2s are generated by IL-25, as opposed to the IL-33-reactive natural ILC2s (nILC2s). These cell types have already been mentioned above: they are present in the intestine and contribute to *N. brasiliensis* expulsion. Similarly, Huang et al. showed that local lung iILC2s helped expel *N. brasiliensis* from the lung; significantly, the iILC2s expressed high levels of KLRG1. KLRG1^+^ iILC2s also provide partial protection from *Candida albicans* infection^141^. Thus, KLRG1 is associated with iILC2-mediated protection from infection. As mentioned above, such KLRG1 expression by iILC2s appears to be downregulated by PD-1 since PD-1 deficiency enhances the ability of iILC2s to expel *N. brasiliensis* from the lungs^132^.

It should be noted that Huang et al. performed in vitro experiments that showed that KLRG1^+^ iILC2s differentiate into nILC2s and ILC3-like cells^141^. Thus, KLRG1^+^ iILC2s may be transient ILC progenitors recruited by infection and inflammation into the lung and intestine, where they then develop into nILC2-like cells and ILC3-like cells^141^. This is supported by a study that showed that KLRG1^+^ ILC precursors (defined as lineage-CD117^+^CRTH2^–^ cells) from human blood differentiate into ILC1s, ILC2s, and ILC3s in vitro depending on the input signals^144^.

Another study suggested that KLRG1^+^ ILC2s may help block allergies *via* ILCreg-like functions^145^. Thus, Golebski et al. showed that KLRG1^+^ ILC2s from human blood produced IL-10 when they were stimulated in vitro with IL-33 and retinoic acid, whereas KLRG1^-^ ILC2s did not exhibit this activity^145^. Moreover, when cultured with nasal epithelium, KLRG1^+^ ILC2s (but not KLRG1^-^ ILC2s) protected the epithelium from the destructive effects of grass-pollen allergen in an IL-10-dependent manner. Similarly, when KLRG1^+^ ILC2s were co-cultured with CD4^+^ T cells, they reduced T-cell activation and proliferation in an IL-10-dependent fashion. Moreover, patients with grass-pollen allergy had lower IL-10^+^KLRG1^+^ ILC2 frequencies in the blood than healthy subjects, but these frequencies were restored by immunotherapy^145^. Thus, KLRG1^+^ ILC2s may help to protect against allergies.

The roles of KLRG1 in ILC1s and ILC3s are unclear. Nonetheless, KLRG1 appears to mediate protective functions by ILC2s against infections and allergies. Further studies on the effect of KLRG1 on ILC cytokine production, proliferation, and roles in mucosal diseases are warranted.

## ILC-targeting therapeutic applications: current methodologies and perspectives

**Fig. 4:**
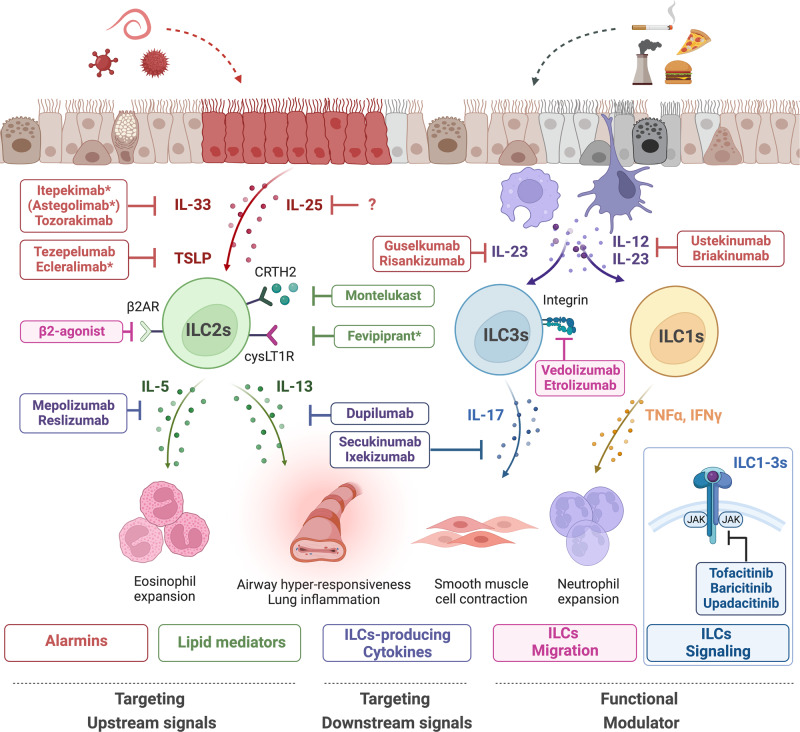
Multifaceted therapies targeting ILCs: modulating migration, signaling, and cytokine pathways. ILCs have the potential to be targeted by therapies to block their inflammatory effects or induce their protective activities. Many biological drugs target alarmins related to the ILC-related inflammatory response. Itepekimab (astegolimab) and tozorakimab (ecleralimab) target the IL-33/ST2 pathway, reducing type 2 inflammation by inhibiting the actions of alarmins. Tezepelumab and ecleralimab, monoclonal antibodies that block the TSLP pathway, are being tested in clinical trials. However, IL-25-targeted drugs have not yet been approved for clinical trials. Mepolizumab and reslizumab are monoclonal antibodies against IL-5, a cytokine produced by ILC2s, which activates IgE production and type 2 inflammation. Dupilumab is a monoclonal antibody that blocks IL-13, a cytokine that promotes eosinophil recruitment. Guselkumab and risankizumab inhibit IL-12, while ustekinumab and briakinumab inhibit IL-23. All of these drugs stop ILC1s and ILC3s from being activated. Some drugs aim to downregulate lipid mediators excreted by ILCs; an example is montelukast, which downregulates CysLT1R. β2AR agonists can reduce lung ILC2 frequencies and cytokine production and ameliorate asthma in a CD4^+^ T-cell-independent manner. To inhibit ILCs directly, some drugs that target the migration and signaling pathways of ILCs have been used. Intestinal ILC3s express high levels of integrin α4β7, a homing receptor necessary for ILC3 accumulation in the intestine. Thus, the anti-α4β7 monoclonal antibody vedolizumab or the anti-β7 monoclonal antibody etrolizumab can be used as inflammation-reducing drugs. JAK/STAT signaling has been shown to play a role in the development and effector functions of every subset of ILCs. Thus, JAK inhibitors such as tofacitinib, baricitinib, and upadacitinib can alleviate ILC-derived inflammation.

Given that ILCs play important roles in mucosal tissue homeostasis and inflammation, many studies have asked whether therapies that aim to downregulate pathogenic ILCs or restore ILC-mediated homeostasis can improve various inflammatory diseases^146^. Therapeutic approaches can be broadly divided according to whether they block inflammatory ILCs or elicit protective ILC activities. Inflammation-blocking approaches, which have received the most research attention, generally employ monoclonal antibodies to block specific cytokines. Below, we will discuss approaches to block ILC-mediated inflammation: the focus will be primarily on therapies that are either in use currently or have been/are being studied in clinical trials and either do or potentially could target ILCs (Fig. 4).

### Drugs that target alarmins

The most common strategy for modulating ILC-dependent pathogenesis is to target (i) the cytokines that act upstream or downstream of ILCs or (ii) the receptors on ILCs that recognize the upstream cytokines. Candidate ILC2 upstream cytokines include the alarmins IL-33, TSLP, and IL-25. These molecules promote the type 2 cytokine production of ILC2s and play a well-established and crucial role in allergic diseases^147^. As a result, most clinical trials on IL-33, TSLP, and IL-25 blockers focus on allergic diseases. The drugs that target the IL-33/ST2 pathway include astegolimab^148,149^, itepekimab^150^, and tozorakimab (formerly MEDI3506)^35^. While these IL-33/ST2-targeting biologics have not yet received FDA approval, some are now in phase 3 clinical trials. The drugs that block the TSLP pathway include tezepelumab, which recently received FDA approval for asthma, and ecleralimab (formerly CSJ117), which is an inhaled drug that is in phase 2 clinical trials (ClinicalTrials.gov Identifier: NCT04882124)^151^. Clinical trials on drugs against IL-25 have not yet been conducted. Moreover, there are also IL-12/23p40 inhibitors, which target a cytokine subunit shared by IL-23 and IL-23 and include ustekinumab^152^ and briakinumab^153^; and IL-23 inhibitors^154^, such as guselkumab and risankizumab^155^.

### Drugs that target ILC-producing cytokines

Candidates target downstream molecules of pathogenic ILC2s, including the type 2 cytokines IL-5 and IL-13. Although type 2 cytokines are produced by pathogenic Th2 cells as well as ILC2s, ILC2s rapidly secrete large amounts of these cytokines soon after the pathogenic trigger^1^. Therefore, several biologics target IL-5 or IL-13 and are already in clinical use and thus could be used to regulate ILC2-mediated pathologies in asthma or allergy. Indeed, dupilumab, an anti-IL-4Rα monoclonal antibody that inhibits the binding of IL-4 and IL-13 to the IL-4Rα receptor, significantly reduced ILC2s in a clinical study on patients with atopic dermatitis^156^. This suggests that anti-IL-5 monoclonal antibodies such as mepolizumab and reslizumab^157,158^, approved for refractory asthma, may also downregulate ILC2s. However, studies on this issue have not yet been conducted. Similarly, the ability of potential inhibitors of ILC1s and/or ILC3s to downregulate the pathogenic activities of these cells is unknown. Candidate drugs are IL-17 inhibitors, which include secukinumab^159^ and ixekizumab^160^.

### Drugs that target lipid mediators

There are also a number of molecules other than alarmins that act upstream of ILC2s and could be targeted to reduce pathogenic ILC2 activity. They include lipid mediators, which activate these cells and thereby promote allergic inflammation; examples are prostaglandin D2 (PGD2) and cysteinyl leukotrienes (cysLTs)^161^. These molecules could be targeted directly or indirectly. An indirect way of targeting PGD2 is to downregulate CRTH2 (chemoattractant receptor-homologous molecule expressed on Th2 cells), which is a PGD2 receptor that is expressed on ILC2s and mediates the PGD2-dependent trafficking of ILC2s that promotes allergies^162^. While strategies targeting the CRTH2-PGD2 axis using the PGD2 antagonist fevipiprant are still under investigation, clinical trials have shown unsatisfactory results to date^162–164^. In contrast, montelukast, a cysLT1 receptor antagonist, is a widely used anti-asthmatic drug^165^ that could potentially act, at least in part, by downregulating the potent ability of cysLTs to activate ILC2s^166^. Studies are needed to verify this idea.

### Drugs that target migration of ILCs

Murine ILC2s express β2AR, and treating IL-33- or allergen-induced murine models of asthma with a β2AR agonist reduces their lung ILC2 frequencies and cytokine production and ameliorates asthma in a CD4^+^ T-cell-independent manner^167^. Thus, β2AR agonists could improve asthma by downregulating ILC2s.

Intestinal ILC3s express high levels of integrin α4β7, and this homing receptor is required for ILC3 accumulation in the intestine^24^. Thus, the anti-α4β7 monoclonal antibody vedolizumab or anti-β7 monoclonal antibody etrolizumab, which are currently being used to treat IBD patients^168–170^, could potentially exert beneficial effects by downregulating ILC3 recruitment to the intestines. This possibility is disputed by a report showing that vedolizumab treatment does not alter the frequencies of ILCs in the blood of patients with Crohn’s disease^171^. However, further research is warranted.

### Drugs that target ILC signaling

ILC activity can be modulated by blocking the signaling pathways that drive ILC activation. These include the Janus kinase (JAK)/signal transducer and activator of transcription (STAT) pathway, which is activated by various cytokines and growth factors in inflammatory diseases^172^. In fact, JAK/STAT signaling contributes to the development and effector functions of all ILC subsets^48,173,174^. Thus, JAK inhibitors could be repurposed to target inflammatory ILCs in mucosal diseases. These inhibitors include (i) tofacitinib, a pan-JAK inhibitor that is approved for IBD^175^; (ii) baricitinib, a JAK1/2 inhibitor that is approved for atopic dermatitis^176^; and (iii) upadacitinib, a JAK1-selective inhibitor that is also approved for atopic dermatitis^177^.

## Concluding remarks

Mucosal organs, including the lungs and digestive tract, are constantly challenged by various stimuli. ILCs are multifunctional immune cells that are permanent residents in these organs; they ceaselessly patrol these tissues and are poised to react instantaneously and strongly when the tissue is compromised. This role means that despite their relatively low frequencies in mucosal tissues, ILCs play important roles in the homeostasis of mucosal organs and the development and progression of mucosal inflammatory diseases. This suggests that ILCs may be an effective therapeutic target for mucosal diseases. Indeed, many approved therapeutic agents and drugs currently in clinical trials have the potential to modulate the function of ILCs in mucosal diseases. Additional drugs for mucosal disorders may be discovered by identifying key interactions (i.e., immune checkpoint interactions) between ILCs and other immune cells and non-immune cells that dictate the immunological outcomes in mucosal tissues. The relatively sparse research to date suggests that DR3-TL1A, PD-1-PD-L1, and KLRG1 may be useful therapeutic targets. Finally, given that ILCs can also play beneficial roles, another potentially effective therapeutic approach is cell therapy. Although this approach is still impeded by technical issues, a few candidate regulatory ILCs have been identified. Further research in this field, and all the other topics mentioned in this review, is warranted.

## References

[1] Vivier E (2018). Innate lymphoid cells: 10 years on. Cell.

[2] Kim J, Ryu S, Kim HY (2021). Innate lymphoid cells in tissue homeostasis and disease pathogenesis. Mol. Cells.

[3] Zhang J (2018). T-bet and Eomes govern differentiation and function of mouse and human NK cells and ILC1. Eur. J. Immunol..

[4] Wagner JA (2020). Stage-specific requirement for eomes in mature NK cell homeostasis and cytotoxicity. Cell Rep..

[5] Chiossone L, Dumas PY, Vienne M, Vivier E (2018). Natural killer cells and other innate lymphoid cells in cancer. Nat. Rev. Immunol..

[6] Entwistle LJ (2020). Pulmonary group 2 innate lymphoid cell phenotype is context specific: determining the effect of strain, location, and stimuli. Front. Immunol.

[7] Monticelli LA (2011). Innate lymphoid cells promote lung-tissue homeostasis after infection with influenza virus. Nat. Immunol..

[8] Turner J-E (2013). IL-9–mediated survival of type 2 innate lymphoid cells promotes damage control in helminth-induced lung inflammation. J. Exp. Med.

[9] Gschwend J (2021). Alveolar macrophages rely on GM-CSF from alveolar epithelial type 2 cells before and after birth. J. Exp. Med.

[10] Meininger I (2020). Tissue-specific features of innate lymphoid cells. Trends Immunol..

[11] Schroeder J-H, Howard JK, Lord GM (2022). Transcription factor-driven regulation of ILC1 and ILC3. Trends Immunol..

[12] Montaldo E, Juelke K, Romagnani C (2015). Group 3 innate lymphoid cells (ILC3s): origin, differentiation, and plasticity in humans and mice. Eur. J. Immunol..

[13] Kim M, Kim CH (2016). Colonization and effector functions of innate lymphoid cells in mucosal tissues. Microbes Infect..

[14] Gasteiger G, Fan X, Dikiy S, Lee SY, Rudensky AY (2015). Tissue residency of innate lymphoid cells in lymphoid and nonlymphoid organs. Science.

[15] Zhong C (2020). Differential expression of the transcription factor GATA3 specifies lineage and functions of innate lymphoid cells. Immunity.

[16] Ghaedi, M. et al. Single-cell analysis of RORalpha tracer mouse lung reveals ILC progenitors and effector ILC2 subsets. *J. Exp. Med.* **217**10.1084/jem.20182293 (2020). 10.1084/jem.20182293 PMC7062532 31816636

[17] Orimo K (2021). Characteristics of tissue-resident ILCs and their potential as therapeutic targets in mucosal and skin inflammatory diseases. Allergy.

[18] Zeis P (2020). In situ maturation and tissue adaptation of type 2 innate lymphoid cell progenitors. Immunity.

[19] Possot C (2011). Notch signaling is necessary for adult, but not fetal, development of RORgammat(+) innate lymphoid cells. Nat. Immunol..

[20] Constantinides MG, McDonald BD, Verhoef PA, Bendelac A (2014). A committed precursor to innate lymphoid cells. Nature.

[21] Erle DJ (1994). Expression and function of the MAdCAM-1 receptor, integrin alpha 4 beta 7, on human leukocytes. J. Immunol..

[22] Deem TL, Cook-Mills JM (2004). Vascular cell adhesion molecule 1 (VCAM-1) activation of endothelial cell matrix metalloproteinases: role of reactive oxygen species. Blood.

[23] Chea S (2015). CXCR6 expression is important for retention and circulation of ILC precursors. Mediators Inflamm.

[24] Kim MH, Taparowsky EJ, Kim CH (2015). Retinoic acid differentially regulates the migration of innate lymphoid cell subsets to the gut. Immunity.

[25] Patman G (2015). Immunology: gut migration of innate lymphoid cells. Nat. Rev. Gastroenterol. Hepatol..

[26] Stier MT (2018). IL-33 promotes the egress of group 2 innate lymphoid cells from the bone marrow. J. Exp. Med..

[27] Corral D (2022). ILC precursors differentiate into metabolically distinct ILC1-like cells during Mycobacterium tuberculosis infection. Cell Rep..

[28] Fuchs A (2013). Intraepithelial type 1 innate lymphoid cells are a unique subset of IL-12- and IL-15-responsive IFN-gamma-producing cells. Immunity.

[29] Luci C (2009). Influence of the transcription factor RORgammat on the development of NKp46+ cell populations in gut and skin. Nat. Immunol..

[30] Klose CS (2013). A T-bet gradient controls the fate and function of CCR6-RORγt+ innate lymphoid cells. Nature.

[31] Hewitt RJ, Lloyd CM (2021). Regulation of immune responses by the airway epithelial cell landscape. Nat. Rev. Immunol..

[32] Mjosberg J, Spits H (2016). Human innate lymphoid cells. J. Allergy Clin. Immunol..

[33] Stephen T. et al. Asthma. *Nat. Rev. Dis. Primers* **1**10.1038/nrdp.2015.25 (2015).

[34] Ham, J., Lim, M., Kim, D. & Kim, H. Y. Memory-like innate lymphoid cells in the pathogenesis of asthma. *Front. Immunol.* **13**10.3389/fimmu.2022.1005517 (2022). 10.3389/fimmu.2022.1005517 PMC9712946 36466877

[35] Ham J, Shin JW, Ko BC, Kim HY (2022). Targeting the epithelium-derived innate cytokines: from bench to bedside. Immune Netw..

[36] Halim TY (2014). Group 2 innate lymphoid cells are critical for the initiation of adaptive T helper 2 cell-mediated allergic lung inflammation. Immunity.

[37] Grotenboer NS, Ketelaar ME, Koppelman GH, Nawijn MC (2013). Decoding asthma: translating genetic variation in IL33 and IL1RL1 into disease pathophysiology. J. Allergy Clin. Immunol..

[38] Tamari M, Tanaka S, Hirota T (2013). Genome-wide association studies of allergic diseases. Allergol. Int..

[39] Liu T (2015). Type 2 innate lymphoid cells: a novel biomarker of eosinophilic airway inflammation in patients with mild to moderate asthma. Respir. Med..

[40] Bartemes KR, Kephart GM, Fox SJ, Kita H (2014). Enhanced innate type 2 immune response in peripheral blood from patients with asthma. J. Allergy. Clin. Immunol..

[41] Kim J (2019). Innate immune crosstalk in asthmatic airways: Innate lymphoid cells coordinate polarization of lung macrophages. J. Allergy Clin. Immunol..

[42] Akar-Ghibril N, Casale T, Custovic A, Phipatanakul W (2020). Allergic endotypes and phenotypes of asthma. J. Allergy Clin. Immunol. Pract..

[43] Kim HY (2014). Interleukin-17-producing innate lymphoid cells and the NLRP3 inflammasome facilitate obesity-associated airway hyperreactivity. Nat. Med..

[44] Estrella, B. et al. Effects of air pollution on lung innate lymphoid cells: review of in vitro and in vivo experimental studies. *Int. J. Environ. Res. Public Health* **16**10.3390/ijerph16132347 (2019). 10.3390/ijerph16132347 PMC6650824 31269777

[45] Kim J (2020). The effect of air pollutants on airway innate immune cells in patients with asthma. Allergy.

[46] Luo J (2019). Resistance to apoptosis underpins the corticosteroid insensitivity of group 2 innate lymphoid cells. J. Allergy Clin. Immunol..

[47] Ploeg EKVD (2021). Steroid-resistant human inflammatory ILC2s are marked by CD45RO and elevated in type 2 respiratory diseases. Sci. Immunol..

[48] Liu S (2018). Steroid resistance of airway type 2 innate lymphoid cells from patients with severe asthma: the role of thymic stromal lymphopoietin. J. Allergy Clin. Immunol..

[49] Moriyama S (2018). β2-adrenergic receptor-mediated negative regulation of group 2 innate lymphoid cell responses. Science.

[50] Rabe KF, Watz H (2017). Chronic obstructive pulmonary disease. Lancet.

[51] Blomme EE (2021). Quantification and role of innate lymphoid cell subsets in Chronic Obstructive Pulmonary Disease. Clin. Transl. Immunology.

[52] Freeman CM (2010). Cytotoxic potential of lung CD8( + ) T cells increases with chronic obstructive pulmonary disease severity and with in vitro stimulation by IL-18 or IL-15. J. Immunol..

[53] Wang Z (2000). Interferon γ Induction of Pulmonary Emphysema in the Adult Murine Lung. J. Exp. Med.

[54] Freeman CM (2014). Human CD56+ cytotoxic lung lymphocytes kill autologous lung cells in chronic obstructive pulmonary disease. PLoS One.

[55] Finch DK (2018). Lung dendritic cells drive natural killer cytotoxicity in chronic obstructive pulmonary disease via IL-15 Ralpha. Am. J. Respir. Crit. Care Med..

[56] Silver JS (2016). Inflammatory triggers associated with exacerbations of COPD orchestrate plasticity of group 2 innate lymphoid cells in the lungs. Nat. Immunol..

[57] Bal SM (2016). IL-1beta, IL-4 and IL-12 control the fate of group 2 innate lymphoid cells in human airway inflammation in the lungs. Nat. Immunol..

[58] Kearley J (2015). Cigarette smoke silences innate lymphoid cell function and facilitates an exacerbated type I interleukin-33-dependent response to infection. Immunity.

[59] De Grove KC (2016). Characterization and quantification of innate lymphoid cell subsets in human lung. PLoS One.

[60] Suzuki M (2017). The cellular and molecular determinants of emphysematous destruction in COPD. Sci. Rep..

[61] Torres A (2021). Pneumonia. Nat. Rev. Dis. Primers.

[62] Stehle C, Hernandez DC, Romagnani C (2018). Innate lymphoid cells in lung infection and immunity. Immunol. Rev..

[63] Dou Y (2015). Influenza vaccine induces intracellular immune memory of human NK cells. PLoS One.

[64] Wills-Karp M, Finkelman FD (2011). Innate lymphoid cells wield a double-edged sword. Nat. Immunol..

[65] Gorski SA, Hahn YS, Braciale TJ (2013). Group 2 innate lymphoid cell production of IL-5 is regulated by NKT cells during influenza virus infection. PLoS Pathog..

[66] Gomes AMC (2021). SARS-CoV2 pneumonia recovery is linked to expansion of innate lymphoid cells type 2 expressing CCR10. Eur. J. Immunol..

[67] Silverstein NJ (2022). Innate lymphoid cells and COVID-19 severity in SARS-CoV-2 infection. Elife.

[68] Van Maele L (2014). Activation of Type 3 innate lymphoid cells and interleukin 22 secretion in the lungs during Streptococcus pneumoniae infection. J. Infect. Dis..

[69] Mowat AM, Agace WW (2014). Regional specialization within the intestinal immune system. Nat. Rev. Immunol..

[70] Yudanin NA (2019). Spatial and temporal mapping of human innate lymphoid cells reveals elements of tissue specificity. Immunity.

[71] Diefenbach A, Gnafakis S, Shomrat O (2020). Innate lymphoid cell-epithelial cell modules sustain intestinal homeostasis. Immunity.

[72] Sonnenberg GF, Fouser LA, Artis D (2011). Border patrol: regulation of immunity, inflammation and tissue homeostasis at barrier surfaces by IL-22. Nat. Immunol..

[73] Sawa S (2011). RORgammat+ innate lymphoid cells regulate intestinal homeostasis by integrating negative signals from the symbiotic microbiota. Nat. Immunol..

[74] Zheng Y (2008). Interleukin-22 mediates early host defense against attaching and effacing bacterial pathogens. Nat. Med..

[75] Sugimoto K (2008). IL-22 ameliorates intestinal inflammation in a mouse model of ulcerative colitis. J. Clin. Invest..

[76] Satoh-Takayama N (2008). Microbial flora drives interleukin 22 production in intestinal NKp46+ cells that provide innate mucosal immune defense. Immunity.

[77] Sonnenberg GF, Monticelli LA, Elloso MM, Fouser LA, Artis D (2011). CD4(+) lymphoid tissue-inducer cells promote innate immunity in the gut. Immunity.

[78] Lindemans CA (2015). Interleukin-22 promotes intestinal-stem-cell-mediated epithelial regeneration. Nature.

[79] Jarade A (2022). Inflammation triggers ILC3 patrolling of the intestinal barrier. Nat. Immunol..

[80] Chun E (2019). Metabolite-sensing receptor Ffar2 regulates colonic Group 3 innate lymphoid cells and gut. Immunity.

[81] Kinnebrew MA (2012). Interleukin 23 production by intestinal CD103(+)CD11b(+) dendritic cells in response to bacterial flagellin enhances mucosal innate immune defense. Immunity.

[82] Longman RS (2014). CX(3)CR1(+) mononuclear phagocytes support colitis-associated innate lymphoid cell production of IL-22. J. Exp. Med..

[83] Takatori H (2009). Lymphoid tissue inducer-like cells are an innate source of IL-17 and IL-22. J. Exp. Med..

[84] Powell N (2012). The transcription factor T-bet regulates intestinal inflammation mediated by interleukin-7 receptor+ innate lymphoid cells. Immunity.

[85] Bhatt B (2018). Gpr109a limits microbiota-induced IL-23 production to constrain ILC3-mediated colonic inflammation. J. Immunol..

[86] Cella M, Otero K, Colonna M (2010). Expansion of human NK-22 cells with IL-7, IL-2, and IL-1β reveals intrinsic functional plasticity. Proc. Natl. Acad. Sci. USA.

[87] Zeng B (2019). ILC3 function as a double-edged sword in inflammatory bowel diseases. Cell Death Dis..

[88] Song C (2015). Unique and redundant functions of NKp46 + ILC3s in models of intestinal inflammation. J. Exp. Med..

[89] Chang Y (2021). Increased GM-CSF-producing NCR(-) ILC3s and neutrophils in the intestinal mucosa exacerbate inflammatory bowel disease. Clin. Transl. Immunol..

[90] Eken A, Singh AK, Treuting PM, Oukka M (2014). IL-23R+ innate lymphoid cells induce colitis via interleukin-22-dependent mechanism. Mucosal Immunol..

[91] Castellanos JG (2018). Microbiota-Induced TNF-like ligand 1 A drives group 3 innate lymphoid cell-mediated barrier protection and intestinal T cell activation during colitis. Immunity.

[92] Bouchery T, Le Gros G, Harris N (2019). ILC2s-trailblazers in the host response against intestinal helminths. Front. Immunol..

[93] Neill DR (2010). Nuocytes represent a new innate effector leukocyte that mediates type-2 immunity. Nature.

[94] Price AE (2010). Systemically dispersed innate IL-13-expressing cells in type 2 immunity. Proc. Natl. Acad. Sci. USA.

[95] Ricardo-Gonzalez RR (2018). Tissue signals imprint ILC2 identity with anticipatory function. Nat. Immunol..

[96] Huang Y (2018). S1P-dependent interorgan trafficking of group 2 innate lymphoid cells supports host defense. Science.

[97] Wang S (2017). Regulatory innate lymphoid cells control innate intestinal inflammation. Cell.

[98] Hutloff A (1999). ICOS is an inducible T-cell co-stimulator structurally and functionally related to CD28. Nature.

[99] Coyle AJ (2000). The CD28-related molecule ICOS is required for effective T cell-dependent immune responses. Immunity.

[100] Toker A (2018). Regulatory T cells in ovarian cancer are characterized by a highly activated phenotype distinct from that in melanoma. Clin. Cancer Res..

[101] Dong C (2001). ICOS co-stimulatory receptor is essential for T-cell activation and function. Nature.

[102] Van DV, Bauer L, Kroczek RA, Hutloff A (2018). ICOS costimulation differentially affects T cells in secondary lymphoid organs and inflamed tissues. Am. J. Respir. Cell Mol. Biol..

[103] Paclik D, Stehle C, Lahmann A, Hutloff A, Romagnani C (2015). ICOS regulates the pool of group 2 innate lymphoid cells under homeostatic and inflammatory conditions in mice. Eur. J. Immunol..

[104] Maazi H (2015). ICOS:ICOS-ligand interaction is required for type 2 innate lymphoid cell function, homeostasis, and induction of airway hyperreactivity. Immunity.

[105] Kamachi F, Isshiki T, Harada N, Akiba H, Miyake S (2015). ICOS promotes group 2 innate lymphoid cell activation in lungs. Biochem. Biophys. Res. Commun..

[106] Iwanaga N (2020). Host immunology and rational immunotherapy for carbapenem-resistant Klebsiella pneumoniae infection. JCI Insight.

[107] Ward-Kavanagh LK, Lin WW, Sedy JR, Ware CF (2016). The TNF receptor superfamily in co-stimulating and co-inhibitory responses. Immunity.

[108] Tian J, Zhang B, Rui K, Wang S (2020). The role of GITR/GITRL interaction in autoimmune diseases. Front. Immunol..

[109] Galle-Treger L (2019). Costimulation of type-2 innate lymphoid cells by GITR promotes effector function and ameliorates type 2 diabetes. Nat. Commun..

[110] Nagashima H (2018). GITR cosignal in ILC2s controls allergic lung inflammation. J. Allergy Clin. Immunol..

[111] Vashist N (2018). Influenza-activated ILC1s contribute to antiviral immunity partially influenced by differential GITR expression. Front. Immunol..

[112] Cavagnero KJ (2019). Unconventional ST2- and CD127-negative lung ILC2 populations are induced by the fungal allergen Alternaria alternata. J. Allergy Clin. Immunol..

[113] Sugamura K, Ishii N, Weinberg AD (2004). Therapeutic targeting of the effector T-cell co-stimulatory molecule OX40. Nat. Rev. Immunol..

[114] Halim TYF (2018). Tissue-restricted adaptive type 2 immunity is orchestrated by expression of the costimulatory molecule OX40L on group 2 innate lymphoid cells. Immunity.

[115] Wu J (2019). Critical role of OX40/OX40L in ILC2-mediated activation of CD4( + )T cells during respiratory syncytial virus infection in mice. Int. Immunopharmacol..

[116] Drake LY, Iijima K, Kita H (2014). Group 2 innate lymphoid cells and CD4 + T cells cooperate to mediate type 2 immune response in mice. Allergy.

[117] Deng T (2020). ILC3-derived OX40L is essential for homeostasis of intestinal Tregs in immunodeficient mice. Cell Mol. Immunol..

[118] McLaren JE (2010). The TNF-like protein 1A-death receptor 3 pathway promotes macrophage foam cell formation in vitro. J. Immunol..

[119] Twohig JP (2012). The death receptor 3/TL1A pathway is essential for efficient development of antiviral CD4(+) and CD8( + ) T-cell immunity. FASEB J..

[120] Valatas V, Kolios G, Bamias G (2019). TL1A (TNFSF15) and DR3 (TNFRSF25): a co-stimulatory system of cytokines with diverse functions in gut mucosal immunity. Front. Immunol..

[121] Prehn JL (2007). The T cell costimulator TL1A is induced by FcgammaR signaling in human monocytes and dendritic cells. J. Immunol..

[122] Ahn YO (2015). Human group3 innate lymphoid cells express DR3 and respond to TL1A with enhanced IL-22 production and IL-2-dependent proliferation. Eur. J. Immunol..

[123] Meylan F (2014). The TNF-family cytokine TL1A promotes allergic immunopathology through group 2 innate lymphoid cells. Mucosal Immunol..

[124] Yu X (2014). TNF superfamily member TL1A elicits type 2 innate lymphoid cells at mucosal barriers. Mucosal Immunol..

[125] Machida, K. et al. Type 2 innate lymphoid cells expressing death receptor 3 are increased in airway of mild atopic asthmatic subject following allergen inhalation challenge. *J. Allergy Clin. Immunol.* **141**10.1016/j.jaci.2017.12.908 (2018).

[126] Li J (2019). Activation of DR3 signaling causes loss of ILC3s and exacerbates intestinal inflammation. Nat. Commun..

[127] Ishida Y, Agata Y, Shibahara K, Honjo T (1992). Induced expression of PD-1, a novel member of the immunoglobulin gene superfamily, upon programmed cell death. EMBO J..

[128] Andrews LP, Yano H, Vignali DAA (2019). Inhibitory receptors and ligands beyond PD-1, PD-L1 and CTLA-4: breakthroughs or backups. Nat. Immunol..

[129] Day CL (2006). PD-1 expression on HIV-specific T cells is associated with T-cell exhaustion and disease progression. Nature.

[130] Pardoll DM (2012). The blockade of immune checkpoints in cancer immunotherapy. Nat. Rev. Cancer.

[131] Yu Y (2016). Single-cell RNA-seq identifies a PD-1(hi) ILC progenitor and defines its development pathway. Nature.

[132] Taylor S (2017). PD-1 regulates KLRG1(+) group 2 innate lymphoid cells. J. Exp. Med..

[133] Seillet C (2016). Deciphering the innate lymphoid cell transcriptional program. Cell Rep..

[134] Vacca P (2019). PD-1 is expressed by and regulates human group 3 innate lymphoid cells in human decidua. Mucosal Immunol..

[135] Tumino N (2019). Presence of innate lymphoid cells in pleural effusions of primary and metastatic tumors: Functional analysis and expression of PD-1 receptor. Int. J. Cancer.

[136] Mariotti FR, Quatrini L, Munari E, Vacca P, Moretta L (2019). Innate lymphoid cells: expression of PD-1 and other checkpoints in normal and pathological conditions. Front. Immunol..

[137] Mallett G, Laurence A, Amarnath S (2019). Programmed cell death-1 receptor (PD-1)-mediated regulation of innate lymphoid cells. Int. J. Mol. Sci.

[138] Helou DG (2020). PD-1 pathway regulates ILC2 metabolism and PD-1 agonist treatment ameliorates airway hyperreactivity. Nat. Commun..

[139] Myklebust JH (2013). High PD-1 expression and suppressed cytokine signaling distinguish T cells infiltrating follicular lymphoma tumors from peripheral T cells. Blood.

[140] Huntington ND (2007). NK cell maturation and peripheral homeostasis is associated with KLRG1 up-regulation. J. Immunol..

[141] Huang Y (2015). IL-25-responsive, lineage-negative KLRG1(hi) cells are multipotential ‘inflammatory’ type 2 innate lymphoid cells. Nat. Immunol..

[142] Mazzurana L (2021). Tissue-specific transcriptional imprinting and heterogeneity in human innate lymphoid cells revealed by full-length single-cell RNA-sequencing. Cell Res..

[143] Blanquart E (2022). Targeting androgen signaling in ILC2s protects from IL-33-driven lung inflammation, independently of KLRG1. J. Allergy Clin. Immunol..

[144] Nagasawa M (2019). KLRG1 and NKp46 discriminate subpopulations of human CD117( + )CRTH2(-) ILCs biased toward ILC2 or ILC3. J. Exp. Med..

[145] Golebski K (2021). Induction of IL-10-producing type 2 innate lymphoid cells by allergen immunotherapy is associated with clinical response. Immunity.

[146] Cobb LM, Verneris MR (2021). Therapeutic manipulation of innate lymphoid cells. JCI Insight.

[147] Matsuyama T (2022). The therapeutic potential for targeting group 2 innate lymphoid cells in asthma. Front. Immunol..

[148] Kelsen SG (2021). Astegolimab (anti-ST2) efficacy and safety in adults with severe asthma: a randomized clinical trial. J Allergy Clin. Immunol..

[149] Yousuf AJ (2022). Astegolimab, an anti-ST2, in chronic obstructive pulmonary disease (COPD-ST2OP): a phase 2a, placebo-controlled trial. Lancet Respir. Med..

[150] Wechsler ME (2021). Efficacy and safety of itepekimab in patients with moderate-to-severe asthma. N. Engl. J. Med..

[151] O’Byrne PM (2022). Development of an inhaled anti-TSLP therapy for asthma. Pulm. Pharmacol. Ther..

[152] Sands BE (2019). Ustekinumab as induction and maintenance therapy for ulcerative colitis. N. Engl. J. Med..

[153] Reich K (2011). A 52-week trial comparing briakinumab with methotrexate in patients with psoriasis. N. Engl. J. Med..

[154] Reich K (2019). Guselkumab versus secukinumab for the treatment of moderate-to-severe psoriasis (ECLIPSE): results from a phase 3, randomised controlled trial. Lancet.

[155] Gordon KB (2018). Efficacy and safety of risankizumab in moderate-to-severe plaque psoriasis (UltIMMa-1 and UltIMMa-2): results from two double-blind, randomised, placebo-controlled and ustekinumab-controlled phase 3 trials. Lancet.

[156] Imai Y, Kusakabe M, Nagai M, Yasuda K, Yamanishi K (2021). Dupilumab effects on innate lymphoid cell and helper T cell populations in patients with atopic dermatitis. JID Innov..

[157] Ortega HG (2014). Mepolizumab treatment in patients with severe eosinophilic asthma. N. Engl. J. Med..

[158] Castro M (2015). Reslizumab for inadequately controlled asthma with elevated blood eosinophil counts: results from two multicentre, parallel, double-blind, randomised, placebo-controlled, phase 3 trials. Lancet Respir. Med..

[159] Langley RG (2014). Secukinumab in plaque psoriasis–results of two phase 3 trials. N. Engl. J. Med..

[160] Gordon KB (2016). Phase 3 trials of ixekizumab in moderate-to-severe plaque psoriasis. N. Engl. J. Med..

[161] Orimo K, Saito H, Matsumoto K, Morita H (2020). Innate lymphoid cells in the airways: their functions and regulators. Allergy Asthma Immunol. Res..

[162] Xue L (2014). Prostaglandin D2 activates group 2 innate lymphoid cells through chemoattractant receptor-homologous molecule expressed on TH2 cells. J. Allergy Clin. Immunol..

[163] Farne H (2021). Effect of CRTH2 antagonism on the response to experimental rhinovirus infection in asthma: a pilot randomised controlled trial. Thorax.

[164] Brightling CE (2021). Effectiveness of fevipiprant in reducing exacerbations in patients with severe asthma (LUSTER-1 and LUSTER-2): two phase 3 randomised controlled trials. Lancet Respir. Med..

[165] Leff JA (1998). Montelukast, a leukotriene-receptor antagonist, for the treatment of mild asthma and exercise-induced bronchoconstriction. N. Engl. J. Med..

[166] Salimi M (2017). Cysteinyl leukotriene E(4) activates human group 2 innate lymphoid cells and enhances the effect of prostaglandin D(2) and epithelial cytokines. J. Allergy Clin. Immunol..

[167] Moriyama S (2018). beta(2)-adrenergic receptor-mediated negative regulation of group 2 innate lymphoid cell responses. Science.

[168] Feagan BG (2013). Vedolizumab as induction and maintenance therapy for ulcerative colitis. N. Engl. J. Med..

[169] Dai B (2021). Dual targeting of lymphocyte homing and retention through alpha4beta7 and alphaEbeta7 inhibition in inflammatory bowel disease. Cell Rep. Med..

[170] Peyrin-Biroulet L (2022). Etrolizumab as induction and maintenance therapy for ulcerative colitis in patients previously treated with tumour necrosis factor inhibitors (HICKORY): a phase 3, randomised, controlled trial. Lancet Gastroenterol. Hepatol..

[171] Forkel M (2019). Distinct alterations in the composition of mucosal innate lymphoid cells in newly diagnosed and established crohn’s disease and ulcerative colitis. J. Crohns Colitis.

[172] Georas SN, Donohue P, Connolly M, Wechsler ME (2021). JAK inhibitors for asthma. J. Allergy Clin. Immunol..

[173] Stabile H (2018). JAK/STAT signaling in regulation of innate lymphoid cells: the gods before the guardians. Immunol. Rev..

[174] Robinette ML (2018). Jak3 deficiency blocks innate lymphoid cell development. Mucosal Immunol..

[175] Sandborn WJ (2017). Tofacitinib as induction and maintenance therapy for ulcerative colitis. N. Engl. J. Med..

[176] Simpson EL (2020). Baricitinib in patients with moderate-to-severe atopic dermatitis and inadequate response to topical corticosteroids: results from two randomized monotherapy phase III trials. Br. J. Dermatol..

[177] Guttman-Yassky E (2020). Upadacitinib in adults with moderate to severe atopic dermatitis: 16-week results from a randomized, placebo-controlled trial. J. Allergy Clin. Immunol..

